# Pilonidal sinus: a comparative study of treatment methods

**Published:** 2014-03-25

**Authors:** I Varnalidis, O Ioannidis, G Paraskevas, D Papapostolou, SG Malakozis, S Gatzos, L Tsigkriki, M Ntoumpara, A Papadopoulou, A Makrantonakis, N Makrantonakis

**Affiliations:** * Plastic Surgical Department, General Regional Hospital ‘George Papanikolaou’. Thessaloniki, Greece; ** First Surgical Department, General Regional Hospital ‘George Papanikolaou’. Thessaloniki, Greece; *** Department of Anatomy, Medical School, Aristotle University of Thessaloniki, Thessaloniki, Greece

**Keywords:** marsupialization, primary closure, open excision, healing time, recurrence

## Abstract

Abstract

Introduction: Pilonidal disease is a very common anorectal problem without a clinical consensus on its optimal management.

Objective: To compare the methods used by our clinic and determine the outcomes in relation to healing, hospitalization time and recurrence.

Materials and Methods: We have studied all the cases of patients with pilonidal sinus that were treated surgically in our clinic from January 1, 1997 to December 31, 1999.

Results: A total of 111 patients were treated of whom 92 (82,8%) were men and 19 (17,2%) were women. Ages ranged from16 to 65 years with an average age of about 25,1 years. Of the 111 patients, 63 were treated with marsupializationand the remaining 48 were treated by excision (29 with open excision and 19 with the primary suture technique). One hundred and two (91,9%) patients were discharged from the hospital after the surgical procedure, while the remaining 9 patients were hospitalized for 24 hours. The healing time for marsupialization was 27,3 days, the primary suture technique was 11,7 days and the open excision method took 46,4 days. Recurrence was observed in 16 patients (14,4%). Recurrence appeared in 4 (6,35%) of the 63 patients subjected to marsupialization, 1 of the 29 patients subjected to open incision, and 11 (57,8%) of the 19 patients subjected to primary closure.

Conclusion: In the absence of inflammation and/or recurrence, marsupialization is the surgical method of choice as it has a low percentage of recurrence and an acceptably short healing period.In apparently large, inflamed and recurrent situations, open excision is preferred.

## Introduction

Pilonidal disease is a very common anorectal problem that most often arises in the hair follicles of the natal cleft of the sacrococcygeal area.Incidence was calculated to be 26 cases per 100.000, affecting males twice as much as females, and is most common in young adults of working age. Men are thought to be at higher risk because of their hirsute nature. Pilonidal sinus is also associated with obesity (37%), sedentary occupation (44%) and local irritation or trauma (34%) [**1**]. A lack of personal hygiene does not appear to contribute, although the Hawthorne effect may have influenced responses where the q factor was entered into profile questionnaire [**1**.**2**]. During the Second World War, pilonidal disease very commonly appeared in jeep drivers, leading to the disease being known as, “jeep disease” [**3**]. 

 Pilonidal disease can appear as an acute abscess along with sinus tract formation. A more complex manifestation can be characterized by chronic or recurrent abscesses with extensive, branching sinus tracts [**4**]. The common form is an acute abscess characterized by the existence of a midline pit in the natal cleft typically identified 4 to 8 cm from the anus. The skin enters the sinus giving the opening a smooth edge. This primary tract leads into a subcutaneous cavity, which contains granulation tissue and usually a nest of hairs that are present in two thirds of cases in men and in one third of those in women and may be seen projecting from the skin opening. Many patients have secondary lateral openings 2 to 5 cm above the midline pit. The skin opening and the superficial portion of the tract are lined with squamous cell epithelium, but the deep cavity and its extensions are not. 

 Today pilonidal sinuses are widely accepted to be acquired abnormalities [**5**,**7**]as a result of the drainage of a hair follicle [**8**] that ruptured in the subcutaneous fat, producing acute or chronic inflammation [**9**] resulting in an abscess or a tract [**7**]. The invasion of the follicle occurs through the expandable orifice of the vestigial scent gland [**10**] and is a result of inflammation and rupture in the subcutaneous fat of the follicle [**10**,**11**]. 

 The management of pilonidal disease depends on its presentation and ranges from simple incision and drainage to a wide excision with extensive reconstructive procedures. There is no clinical consensus on the optimal management of the pilonidal sinus and our objective is to compare the methods used by our clinic and determine the outcomes in relation to healing, hospitalization time and recurrence. We also try to determine the statistical occurrence based on sex and age. 

## Materials and Methods

We have studied all the cases of patients with Pilonidal sinus that were treated surgically in our clinic from January 1, 1997 to December 31, 1999.

The variables studied were:

-Age

-Sex

 -Method of treatment

-Time of hospitalization

-Time of healing

-Recurrence

## Results

Our clinic surgically treated 111 patients presenting with pilonidal sinus from January 1, 1997 to December 31, 1999. Among these 111 patients, 92 (82,8%) were men and 19 (17,2%) were women. Ages ranged from16 to 65 years with an average age of about 25,1 years.

 In our clinic we practice the Open or Primary Suture Excision and marsupialization techniques. There was no inflammation in any of the 111 treated patients.If there was prior inflammation, it was treated first with incision and drainage followed by administration of medication, including antibiotics and non-steroid anti-inflammatory drugs.

 Of the 111 patients, 63 were treated with marsupializationand the remaining 48 were treated by excision (in 29 of them open excision was preferred, while the remaining 19 received the primary suture technique). From the total number of patients, 102 (91,9%) were discharged from the hospital after the surgical procedure, while in the remaining 9 cases, hospitalization for 24 hours was deemed necessary.

**Fig. 1:**
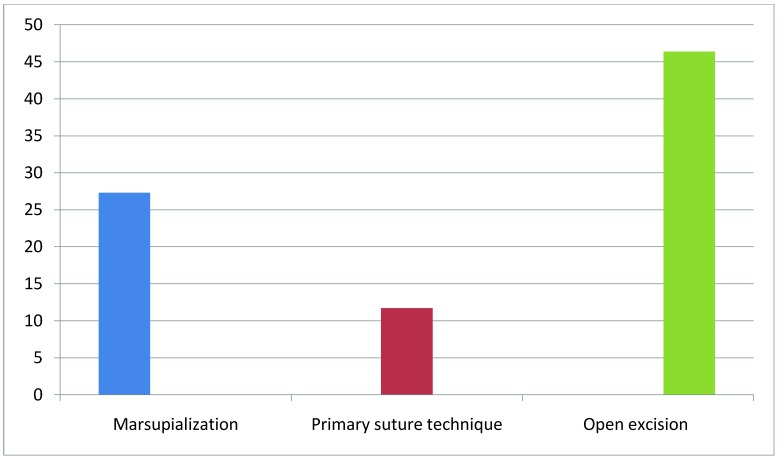
Healing time of the surgical techniques

 The time of healing (**Fig. 1**) of the patients that were subjected to marsupialization peaked at 40 days (average 27,3), in contrast to the patients subjected to the primary suture technique, which peaked at 15 days (average 11,7). The patients that were subjected to the open excision method had a healing time that peaked at 90 days (average 46,4).

**Fig. 2:**
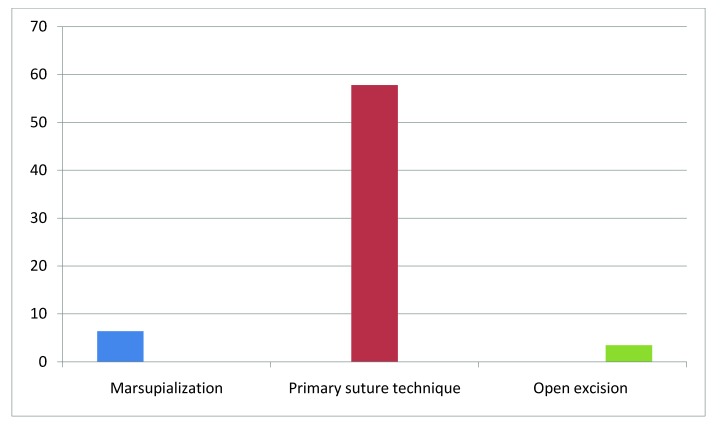
Recurrence rate of the surgical techniques

 Recurrence of sinus disease (**Fig. 2**) was observed in 16 patients (14,4%). Four (6,35%) of the 63 patients subjected to marsupialization experienced recurrence. Of the 29 patients subjected to open incision, there was only 1 (3,45%) recurrence, while 11 (57,8%) of the 19 patients subjected to primary closure experienced recurrence.

## Discussion

Pilonidal disease affects men [**12**,**14**] between 16-25 years of age most often. Usually it is associated with obese [**15**,**17**] and hirsute individuals who experience profuse sweating and have a sedentary lifestyle [**3**,**18**]. The treatment of pilonidal disease is mostly surgical. The most commonly used procedures today are: simple incision, excision, plastic surgery techniques, marsupialization and fistulotomy.

 Simple incision implies a midline incision through the mouths of the pits and is effective in those cases of so-called raphe cannulization where infection spreads from pit to pit [**11**,**19**]. After unroofing the tract it is cleaned and drained. The final cure is done after the end of inflammation [**20**,**22**].This is usually reserved for acute infective swelling. Recurrence is frequent and is mostly used in acute situations where relief of pain is urgent.

 Excision is used for chronic and recurrent pilonidal sinuses. Excision of all involved skin and subcutaneous tissue may be necessary for definitive treatment. These wounds may then be managed openly, with healing by secondary intention, allowing the wound to granulate, or is closed by primary suture [**23**,**24**].Laying the sinus open permits adequate drainage. The healing requires more time, but has lower recurrence [**25**].In the primary suture the pilonidal sinus is excised and the wound sutured by using deep tension sutures tied over a gauze dressing. The advantages are quicker healing and an early return to work, albeit with higher recurrence when compared to the open technique [**26**,**27**].

 Plastic surgery techniques that include these procedures do not only cover the wound but also, in theory, fatten the natal cleft, reduce hair accumulation, mechanical irritation and risk of recurrence [**1**,**12**]. Various kinds of flaps have been used: 1-2 skin flaps, fasciocutaneous flaps like the V-Y flap (for recurrent and complicated sinus disease) and rhomboid excision and the Limberg flap [**28**]. Moreover, the Karydakis flap [**29**] achieves asymmetric closure of the pilonidal wounds by avoiding to place the wound in the midline at the depth of the natal cleft and also flattens the cleft reducing hair accumulation and mechanical irritation [**29**,**30**] in order to decrease recurrence.

 Marsupializationinvolves milossis and opening up of the cavity [**9**,**31**,**32**]. After excision of the sinus front and lateral tracts the cavity is scrubbed to remove hair and granulation tissue. Then, the skin flaps are sutured to the presacral fascia and the wound healing is done by secondary intention [**11**,**33**]. It is vital to have a strong front tract in order to succeed.

 Fistulotomy involves milossis of the cavernous resource, opening up, removal of hair and scrubbing of granulation tissue and healing by secondary intention [**34**,**35**].

For our cases, we used incision and drainage of the abscessed bladders followed by excision (Open or with Primary Closure) and marsupialization [**36**].Clear criteria for selecting the treatment method do not exist [**37**]. Nevertheless, it is emphasized that Primary Closure should be used in small, uncomplicated bladders [**38**] and the open excision in larger bladders [**39**].

 After incision and drainage of the bladder has been performed, and after inflammation has subsided, a permanent treatment can be applied. Based on our 111 observed surgical cases, marsupialization is the surgical method of choice as it had a low percentage of recurrence and an acceptably short healing period [**37**].It should be noted that selecting marsupialization as a treatment method presupposes the absence of inflammation and that the case is not a recurrence [**40**]. In apparently large, inflamed and recurrent situations, we should prefer the Open Excision, where the healing time is longer but the percentage of success is greater [**41**].
